# Effect and mechanism of repetitive transcranial magnetic stimulation of angular gyrus on patients with consciousness disorder

**DOI:** 10.3389/fneur.2026.1868819

**Published:** 2026-06-17

**Authors:** Linghui Dong, Hui Li, Kaiyue Han, Zhiqing Tang, Xingxing Liao, Junzi Long, Xinyue Liu, Tao Lin, Hao Zhang

**Affiliations:** 1 Qilu Hospital of Shandong University, Jinan City, Shandong, China; 2 University of Health and Rehabilitation Sciences, Qingdao City, Shandong, China; 3 Beijing Bo'ai Hospital, China Rehabilitation Research Center, Beijing, China; 4 Department of Rehabilitation Medicine, Capital Medical University Xuanwu Hospital, Beijing, China

**Keywords:** angular gyrus, coma recovery scale–revised, explainable machine learning, prolonged disorders of consciousness, repetitive transcranial magnetic stimulation, resting-state fMRI

## Abstract

**Objective:**

To determine whether high-frequency rTMS of the left angular gyrus improves behavioral signs of consciousness in prolonged disorders of consciousness (pDoC) and to identify network mechanisms and predictors of response.

**Methods:**

In a randomized, double-blind, sham-controlled crossover trial, patients with pDoC received 3 weeks of 20-Hz rTMS over the left angular gyrus and 3 weeks of sham stimulation in counterbalanced order, separated by a 1-week washout. Consciousness was evaluated using the Coma Recovery Scale–Revised (CRS-R). Resting-state fMRI was acquired at baseline and after each period to quantify network-level changes. Explainable machine-learning models related stimulation-induced network changes and baseline features to behavioral improvement.

**Results:**

Forty patients completed the trial. Behavioral improvement occurred more frequently during active than sham stimulation (*p* < 0.05), predominantly in visual, motor, and auditory domains. Explainable analyses indicated that post-rTMS recovery within the default mode network (DMN) and the subcortical network (SCN) best characterized responders (random forest AUC = 0.861). Stronger baseline within-DMN functional connectivity predicted greater likelihood of improvement (support vector machine AUC = 0.972).

**Conclusion:**

Left angular gyrus rTMS improved consciousness-related behaviors in a subset of pDoC patients, potentially mediated by DMN–SCN network reconfiguration; patients with stronger baseline within-DMN connectivity were more likely to benefit.

**Clinical trial registration:**

https://www.chictr.org.cn/index.html, identifier ChiCTR2300069980.

## 1 Introduction

Prolonged disorders of consciousness (pDoC) are among the most challenging clinical conditions after severe acquired brain injury. Patients may present with unresponsive wakefulness syndrome or minimally conscious state. The central disturbance is not merely a reduction in wakefulness, but a disruption of the relationships among arousal, sensory input, motor output, and conscious content. With advances in emergency care and critical care medicine, an increasing number of patients with severe brain injury survive the acute phase. However, some subsequently enter a prolonged disorder of consciousness, placing sustained pressure on clinical rehabilitation, family care, and medical decision-making (1). At present, therapeutic options for these patients remain limited. Pharmacological treatments, rehabilitation training, and neuromodulation have all been explored to promote recovery of consciousness, but treatment effects vary considerably and patient responses are often inconsistent (2). Therefore, improving consciousness-related behaviors within a relatively safe and reproducible intervention framework, while clarifying the underlying mechanisms of brain network recovery, remains an important issue in rehabilitation research for disorders of consciousness.

In clinical practice, diagnosis and assessment of treatment response in disorders of consciousness still rely mainly on behavioral evaluation. The Coma Recovery Scale-Revised (CRS-R) remains one of the most widely used standardized instruments. The CRS-R captures residual responses across auditory, visual, motor, oromotor/verbal, communication, and arousal domains, and helps distinguish unresponsive wakefulness syndrome from minimally conscious state (3). However, behavioral responses in patients with pDoC are often fluctuating. A single assessment may be affected by fatigue, arousal level, muscle tone, sensory impairment, and the timing of evaluation. Recent work on the interpretation of CRS-R change has emphasized the need to distinguish true improvement from spontaneous fluctuation. This is particularly important in interventional studies, where the clinical meaning of subscale changes should be considered rather than relying solely on total score changes (4). These issues also indicate that behavioral scales alone cannot fully explain the neural mechanisms underlying treatment response. Brain functional assessment is therefore needed to improve mechanistic understanding of therapeutic effects.

In recent years, functional neuroimaging has provided an important complement to the diagnosis, prognostic assessment, and mechanistic study of pDoC. Functional magnetic resonance imaging (fMRI), electroencephalography, and positron emission tomography can help identify residual brain function that may not be detected by conventional bedside assessment. This is especially relevant in patients with limited behavioral responses or impaired motor output (5). Current evidence suggests that recovery of consciousness does not depend only on activation of a single local brain region. Rather, it relies on coordinated integration among the default mode network (DMN), frontoparietal control network (FPCN), sensorimotor network (SMN), thalamocortical circuits, and subcortical structures (6). Among these approaches, resting-state fMRI can characterize spontaneous brain activity and network connectivity without requiring task cooperation, making it particularly suitable for studies involving patients with pDoC (7).

Repetitive transcranial magnetic stimulation (rTMS) is a non-invasive neuromodulation technique that can modulate cortical excitability and remote network activity through time-varying magnetic fields. It has been investigated as a potential rehabilitative intervention for patients with disorders of consciousness. Previous systematic reviews and meta-analyses suggest that rTMS may improve CRS-R scores in some patients with disorders of consciousness. However, existing studies generally have small sample sizes, and stimulation targets, frequencies, treatment duration, and patient selection criteria remain heterogeneous. The overall quality of evidence therefore requires further improvement (8). Some studies have also combined rTMS with other peripheral or central stimulation approaches, suggesting that neuromodulation may influence arousal and consciousness-related networks through multiple pathways. Nevertheless, these effects still need to be confirmed in more rigorously controlled studies (9). Accordingly, rTMS studies should not only evaluate behavioral improvement, but also assess concurrent brain network changes and identify which patients are more likely to benefit from stimulation.

Target selection is a key issue in rTMS intervention for pDoC. Previous studies have largely focused on the dorsolateral prefrontal cortex and the primary motor cortex. However, these targets do not fully cover posterior association cortices that are closely related to conscious integration. The posterior parietal cortex participates in sensory integration, spatial attention, and higher-order cognitive processing. A recent pilot study of parietal rTMS in patients with pDoC suggested that this region may be a promising intervention target beyond traditional prefrontal and motor cortical sites (10). The angular gyrus, located in the posterior part of the inferior parietal lobule, is an important heteromodal association region linking attention, semantic processing, social cognition, and the default mode network. Its function is not restricted to a single cognitive domain, but is closely related to interactions among multiple large-scale brain networks (11). From a cognitive neuroscience perspective, the angular gyrus is also thought to support the online integration and buffering of multisensory and temporally extended information. This functional profile suggests a potential bridging role between external sensory input, internal representation, and higher-level cognitive integration (12). Therefore, targeting the left angular gyrus with high-frequency rTMS has a neuroanatomical and functional network rationale. However, whether stimulation of the left angular gyrus can improve consciousness-related behaviors in patients with pDoC, and whether its effects are associated with reconfiguration of the default mode network, thalamocortical circuits, and subcortical networks, remains to be further examined.

Another unresolved issue is the interindividual variability in treatment response. Patients with pDoC have heterogeneous etiologies, lesion distributions, and degrees of residual network integrity. Even with the same stimulation protocol, clinical responses may differ substantially. In recent years, machine learning has been used to integrate multidimensional clinical and neuroimaging data to improve diagnostic and prognostic prediction (13). However, in medical research, model performance alone is insufficient for explaining disease mechanisms. If a model cannot indicate which features drive its predictions, its clinical interpretability and translational value are limited. Explainable machine learning provides a methodological approach to this problem. It can preserve predictive performance while estimating the contribution of different imaging features to individual or group-level predictions, thereby helping researchers reconnect model outputs with medical mechanisms and brain network function (14). In disorders of consciousness, such methods are particularly suitable for exploring the relationships among baseline brain network status, stimulation-induced network changes, and behavioral improvement.

Based on this background, the present study used a randomized, double-blind, sham-controlled crossover design to evaluate the effects of 20 Hz rTMS over the left angular gyrus on consciousness-related behaviors in patients with pDoC. By combining CRS-R assessment, resting-state fMRI network metrics, and explainable machine learning, this study aimed to address two questions. First, whether high-frequency rTMS of the left angular gyrus promotes consciousness-related behavioral improvement more effectively than sham stimulation. Second, whether brain network changes during treatment and baseline network features before treatment can help explain or predict treatment response. Through this design, we sought to move beyond clinical efficacy observation and provide evidence for the mechanism and individualized application of left angular gyrus rTMS from the perspective of the DMN, FPCN, and related functional connectivity.

## 2 Materials and methods

### 2.1 Study design and oversight

**Figure 1:**
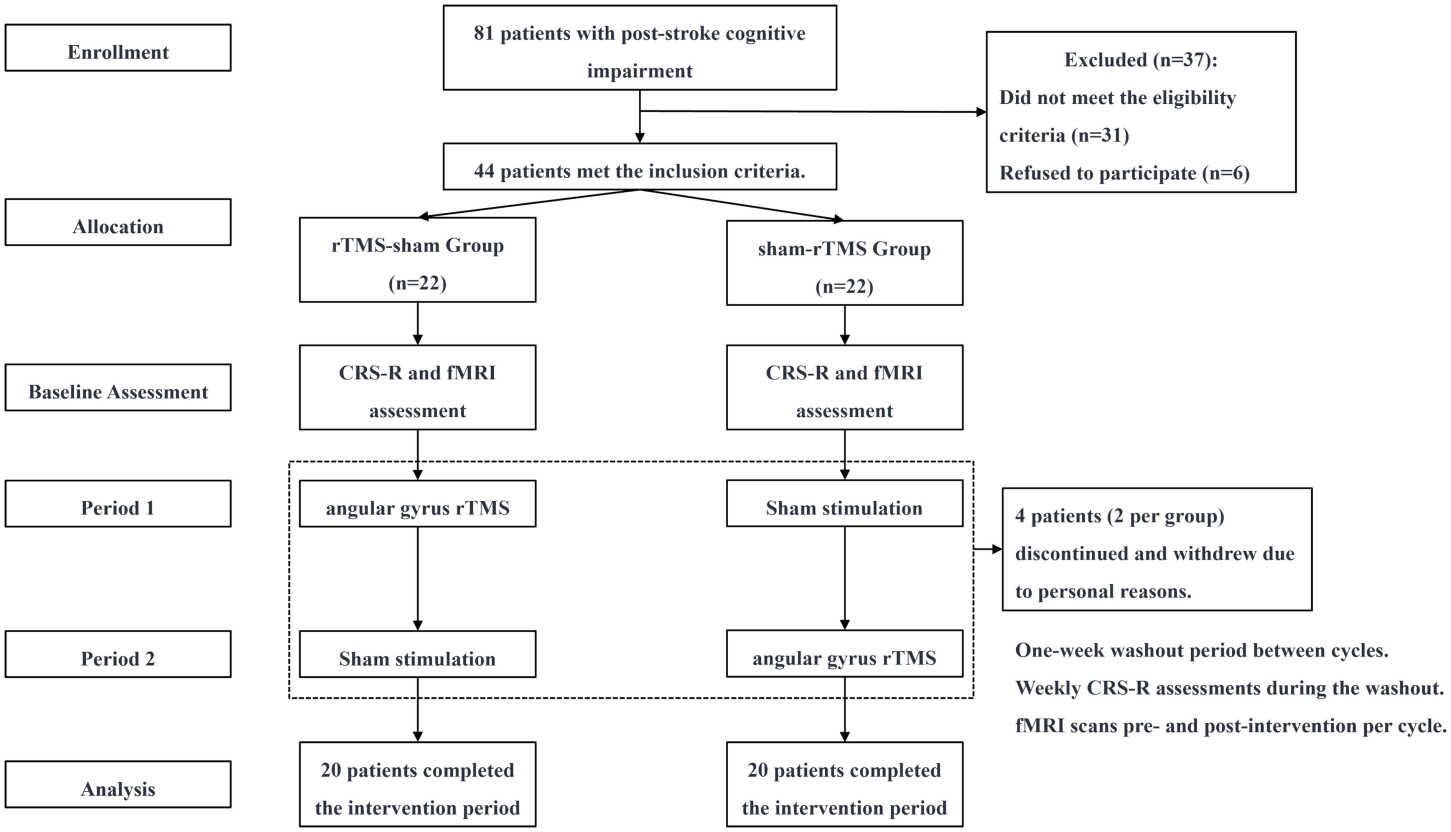
Trial flowchart and crossover study design. Eighty-one patients were screened for eligibility; 37 were excluded (31 did not meet inclusion criteria and 6 declined participation), leaving 44 participants who were randomized to one of two sequences: rTMS–sham (*n* = 22) or sham–rTMS (*n* = 22). All participants underwent baseline assessments including the Coma Recovery Scale–Revised (CRS-R) and resting-state fMRI prior to Period 1. In Period 1, participants received either high-frequency rTMS targeting the left angular gyrus or sham stimulation, followed by a 1-week washout with weekly CRS-R assessments, and then crossed over to the alternate condition in Period 2. Resting-state fMRI was acquired pre- and post-intervention in each period. Four participants (two per sequence) discontinued the study for personal reasons, and 40 participants completed both intervention periods and were included in the final analyses. CRS-R, Coma Recovery Scale–Revised; fMRI, functional magnetic resonance imaging; rTMS, repetitive transcranial magnetic stimulation.

This study was a randomized, double-blind, sham-controlled crossover trial conducted in accordance with the Declaration of Helsinki and approved by the Ethics Committee of the China Rehabilitation Research Center (No. 2023–009-01). The trial was registered in the Chinese Clinical Trial Registry (ChiCTR2300069980). Patients were recruited at the China Rehabilitation Research Center between April 2023 and April 2025. A pilot study involving 8 patients was performed to determine the sample size required for efficacy validation. Using CRS-R as the primary outcome measure, sample size was calculated with a power of 1-*β* = 0.9, significance level *α* = 0.05, and dropout rate of 20%. After 3 weeks of intervention, the CRS-R scores in the rTMS group improved by 0.75 ± 0.5 points, while those in the sham stimulation group improved by 0.25 ± 0.5 points. Independent samples t-test showed that 22 patients were needed in each group. Written informed consent was obtained from each participant or their legal representative. The study design and recruitment flowchart are shown in Figure 1.

### 2.2 Participants

Eligible participants met all of the following criteria: (1) diagnosis of unresponsive wakefulness syndrome/vegetative state or minimally conscious state based on behavioral assessment with the CRS-R; (2) disease duration ≥28 days and <1 year, with a stable clinical condition and first-ever onset; (3) age 18–75 years; and (4) informed consent from family/legal representative.

Exclusion criteria were: severe medical comorbidities (e.g., heart failure, renal failure, acute lung injury, acute pulmonary infection) or severe complications (e.g., severe hydrocephalus); history of psychiatric disease or alcohol/drug abuse; neuromodulation within the past 3 months; intracranial metal foreign body or other contraindications to rTMS; seizure within the past month; skull repair/defect at the stimulation target; or planned surgery in the near term.

### 2.3 Randomization, masking, and crossover procedure

Patients were stratified by level of consciousness (MCS or UWS) and allocated by a random number table to one of two sequences: rTMS→sham or sham→rTMS. Each intervention period lasted 3 weeks and the two periods were separated by a 1-week washout. Participants and family members, outcome assessors, and statisticians were blinded to allocation.

Withdrawal/termination criteria included clinical changes leading to failure to meet inclusion criteria or meeting any exclusion criterion, inability to continue for personal reasons, or withdrawal requested by the patient/family.

### 2.4 rTMS and sham stimulation

rTMS was delivered using a stimulator equipped with a figure-of-eight D70 air-film coil. The target was the left angular gyrus localized at P5 using the international 10–20 EEG system (15). Resting motor threshold (RMT) was determined as the minimum intensity that elicited contraction of the contralateral first dorsal interosseous muscle in at least 5 of 10 trials.

During the active period, stimulation was delivered at 80% RMT, 20 Hz, 3,200 pulses per session for 3 weeks (5 days/week, 1 session/day). During the sham period, stimulation was delivered at the same target and schedule using a sham coil identical in appearance but without an effective magnetic field. Standardized rehabilitation (2 h/day; physical and occupational therapy) was maintained throughout, and concomitant medications and routine rehabilitation regimens were kept unchanged after enrollment.

### 2.5 Clinical outcome assessment

Consciousness was assessed using the CRS-R (six subscales: auditory, visual, motor, oromotor/verbal, communication, and arousal; total score 0–23). Given the difficulty of recovery in pDoC, treatment response was defined as an improvement of ≥1 point in any CRS-R subscale, and the primary endpoint was the number of patients showing CRS-R improvement during each intervention period. Two trained physicians independently assessed CRS-R at baseline and after each week of 5 treatment sessions.

### 2.6 Brain network assessment (resting-state fMRI)

All participants underwent structural (T1-weighted) and resting-state BOLD MRI at two time points within each crossover period: baseline and end of period for both the active rTMS and sham conditions (Philips Ingenia 3.0 T, Philips Healthcare, The Netherlands). Image acquisition and preprocessing followed a standardized, reproducible pipeline, with full acquisition parameters, preprocessing steps, and quality-control criteria.

#### 2.6.1 MRI acquisition

All participants underwent structural (T1-weighted) and resting-state BOLD MRI at baseline and at the end of each intervention period (active rTMS and sham) on a Philips Ingenia 3.0 T scanner (Philips Healthcare, The Netherlands). Resting-state BOLD parameters were: TR 2000 ms, TE 30 ms, flip angle 90°, field of view 224 mm, slice thickness 3.5 mm, inter-slice gap 0.85 mm, 32 slices, matrix 64 × 64. For each time point, three consecutive resting-state runs (8 min each) were acquired. Earplugs/headphones and secure head restraints were used to reduce acoustic noise and minimize head motion.

#### 2.6.2 Preprocessing

Preprocessing was conducted in MATLAB 2023a using the RestPlus toolbox. For each run, data were converted from DICOM to NIfTI format and the first 10 volumes were discarded. Images then underwent slice-timing correction and rigid-body realignment for head-motion correction. Structural T1 images were segmented using DARTEL and functional images were normalized to MNI space with resampling to 3 × 3 × 3 mm^3^. Linear detrending and temporal band-pass filtering (0.01–0.08 Hz) were applied. Nuisance regression included Friston-24 motion parameters and signals from white matter and cerebrospinal fluid. To reduce spatial blurring across neighboring regions and inflation of inter-regional correlations, spatial smoothing was applied only for ALFF computation (Gaussian kernel, 6-mm FWHM). In contrast, DC, FC, and ReHo were derived from unsmoothed data.

#### 2.6.3 Three-run integration and motion-quality control

Repeated acquisitions were used to improve measurement stability. Motion quality control was implemented at the time-point (volume) level across the three runs. A volume was considered motion-contaminated if displacement exceeded 3 mm translation or 3° rotation. For each time point, signals were combined across runs as follows:

- If one run was motion-contaminated at a given time point, the final signal for that time point was computed as the mean of the remaining two qualified runs.
- If two runs were motion-contaminated at the same time point, the final signal was taken from the single remaining qualified run.
- If all three runs were motion-contaminated at the same time point, the dataset was excluded from further analysis (no qualified signal available).

This approach maximized the use of valid data while ensuring that motion-contaminated volumes did not bias downstream metrics.

#### 2.6.4 Derived variables for statistical analyses

For intervention-related analyses, within-subject change (*Δ*) in each metric was computed from baseline to end-of-period for both active rTMS and sham periods, and Δ values were compared between conditions. For responder analyses, baseline metrics were compared between patients who did versus did not show behavioral improvement during the rTMS period. Multiple-comparison control and inferential procedures are described in the main Statistical Analysis section.

#### 2.6.5 Power-264 atlas description and ROI coordinates with AAL anatomical labels

Network analyses were conducted using predefined regions of interest (ROIs) derived from the Power-264 atlas, which consists of 236 nodes assigned to 13 large-scale brain networks, including the DMN, FPCN, cingulo-opercular network (CON), and sensorimotor network (SMN), among others (16). A description of the Power-264 atlas and the MNI coordinates and AAL-based anatomical labels of all ROIs used in this study are provided in Supplementary material 1.

For each node, we quantified complementary markers of intrinsic function: ALFF to index spontaneous neural activity, degree centrality (DC) to reflect whole-brain network importance, and regional homogeneity (ReHo) to capture local functional synchronization. Pairwise functional connectivity (FC) was computed to characterize functional coupling between nodes. These metrics were used to (i) compare within-subject network changes between the active rTMS and sham periods and (ii) examine baseline network differences between patients who did versus did not show behavioral improvement during rTMS.

### 2.7 Explainable machine learning

Two explainable machine-learning models were built with behavioral improvement during the rTMS period as the outcome: (1) models using stimulation-period changes in network features to identify recovery-linked network reconfiguration; and (2) models using baseline network features to identify likely responders. Features were Z-scored, and data were split into training (70%) and testing (30%) sets with proportional allocation of responders. Feature selection used LASSO with five-fold cross-validation and grid search to determine the optimal regularization parameter. Nine classifiers were evaluated: artificial neural network, support vector machine (SVM), k-nearest neighbors, decision tree, LightGBM, gradient boosting, extra trees, random forest, and XGBoost. Model performance was primarily assessed using AUC with five-fold cross-validation in the training set, followed by testing in the held-out test set; SHAP was used to interpret feature importance.

### 2.8 Statistical analysis

Analyses were performed in Python 3.12. Continuous variables are reported as mean ± SD and categorical variables as counts and percentages. Normality was assessed using the Shapiro–Wilk test; between-group comparisons used independent-samples t tests or Mann–Whitney U tests as appropriate. Categorical variables were compared using χ^2^ tests. All tests were two-sided with *p* < 0.05 considered significant, and false discovery rate correction was applied for brain network metrics.

## 3 Results

During recruitment, 81 patients with pDoC were screened. Thirty-one did not meet the eligibility criteria and 6 declined participation. In total, 44 patients were enrolled and randomized to the rTMS–sham or sham–rTMS sequence; four participants withdrew during the trial for personal reasons (two in each group). Ultimately, 40 patients completed both intervention periods (Figure 1). Baseline demographic and clinical characteristics are summarized in Table 1. No rTMS-related adverse events were observed throughout the study.

**Table 1:** Baseline characteristics of participants.

Characteristics	rTMS-sham group (*n* = 20)	sham-rTMS group (*n* = 20)	*p*-value
Age, years	51.2 ± 9.85	55.65 ± 8.91	0.12
Male, *n* (%)	12 (60)	16 (80)	0.30
Etiology, *n* (%)			0.59
TBI	10 (50)	7 (35)	
Intracerebral hemorrhage	9 (45)	11 (55)	
Cerebral infarction	1 (5)	2 (10)	
Time since injury, days	102.5 ± 62.3	114.5 ± 58.8	0.38
Diagnosis, *n* (%)			0.48
MCS	9 (45)	8 (40)	1.00
UWS	11 (55)	12 (60)	
Baseline CRS-R Score	7.8 ± 4.7	7.7 ± 4.3	0.99

TBI, Traumatic Brain Injury. MCS, Minimally Conscious State. UWS, Unresponsive Wakefulness Syndrome. CRS-R, Coma Recovery Scale-Revised. Group A, rTMS first, followed by sham stimulation; Group B, sham stimulation first, followed by rTMS. In the rTMS–sham group, age ranged from 37 to 63 years, time since injury ranged from 33 to 282 days, and baseline CRS-R scores ranged from 2 to 15. In the sham–rTMS group, age ranged from 35 to 66 years, time since injury ranged from 51 to 293 days, and baseline CRS-R scores ranged from 2 to 14.

### 3.1 CRS-R improvement rate

**Figure 2:**
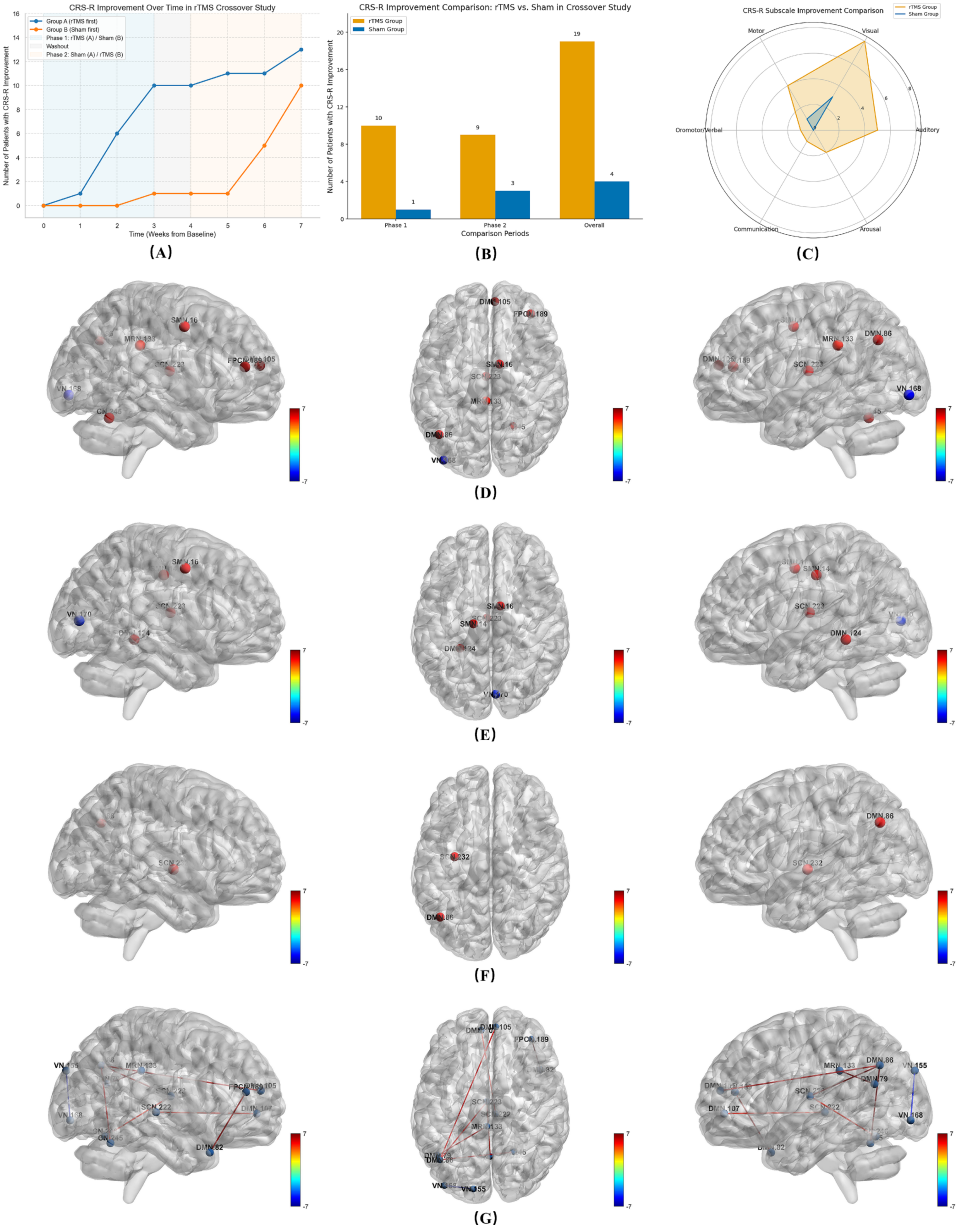
Behavioral and network-level effects of left angular gyrus rTMS in a randomized sham-controlled crossover trial. **(A)** Cumulative number of patients showing behavioral improvement on the Coma Recovery Scale–Revised (CRS-R) over time in the two sequence groups (Group A: rTMS → sham; Group B: sham → rTMS) across two 3-week periods separated by a 1-week washout. Improvement was defined as a ≥ 1-point increase in any CRS-R subscale relative to the baseline of the corresponding period. **(B)** Number of patients with CRS-R improvement during Phase 1, Phase 2, and overall, comparing active rTMS versus sham periods (*p* < 0.05). **(C)** Distribution of improved CRS-R subscales during active versus sham periods, indicating that gains were mainly observed in visual, motor, and auditory domains. **(D–G)** Resting-state fMRI network changes comparing within-subject period-related changes during rTMS versus sham. Analyses used 236 ROIs across 13 large-scale networks derived from the Power-264 parcellation; multiple comparisons across network metrics were controlled using FDR (*p* < 0.05). **(D)** ALFF: greater increases during rTMS in SMN.16, DMN.86 (stimulated left angular gyrus), DMN.105, MRN.133, FPCN.189, SCN.223, and CN.245, with a decrease in VN.168. **(E)** Degree centrality (DC): increases during rTMS in DMN.86 and SCN.232. **(F)** Regional homogeneity (ReHo): increases during rTMS in SMN.14, SMN.16, DMN.124, and SCN.223, with a decrease in VN.170. **(G)** Functional connectivity (FC): increases during rTMS in DMN.86–CN.245, DMN.86–DMN.105, DMN.86–SCN.223, SCN.223–CN.246, DMN.79–MRN.133, DMN.82–FPCN.189, and DMN.107–SCN.222, and a decrease in VN.155–VN.168. For brain maps, warm colors indicate greater increases and cool colors indicate greater decreases during rTMS versus sham; lines denote FC differences (red, increased; blue, decreased). Network abbreviations: DMN, default mode network; FPCN, frontoparietal control network; CON, cingulo-opercular network; SMN, sensorimotor network; MRN, memory retrieval network; SCN, subcortical network; CN, cerebellar network; VN, visual network.

In the first period, 10/20 patients in the rTMS–sham group showed improvement in CRS-R scores relative to baseline, compared with 1/20 patients in the sham–rTMS group (*p* < 0.05; Figure 2A). In the second period, 3/20 patients in the rTMS–sham group improved, whereas 9/20 patients in the sham–rTMS group improved (*p* < 0.05; Figure 2A), consistent with the crossover sequence effect. Across both periods, improvement occurred more frequently during active rTMS than during sham stimulation (19/40 vs. 4/40 intervention periods; *p* < 0.05; Figure 2B). Improvements were mainly concentrated in the visual, motor, and auditory domains (Figure 2C). The detailed CRS-R subscale scores of all patients in both groups at baseline and post-treatment across the two cycles are provided in Supplementary material 2.

Because the sample size was insufficient for formal subgroup analyses by diagnosis or etiology, these findings are reported descriptively. Among the 20 patients in the rTMS–sham group, 10 showed CRS-R improvement during the rTMS period. Of these, 7 were patients with MCS, including 4 with TBI and 3 with intracerebral hemorrhage, and 3 were patients with UWS, including 2 with TBI and 1 with intracerebral hemorrhage. The 3 patients who showed CRS-R improvement during the sham period were all patients with MCS, including 2 with TBI and 1 with intracerebral hemorrhage. Among the 20 patients in the sham–rTMS group, 9 showed CRS-R improvement during the rTMS period. Of these, 6 were patients with MCS, including 2 with intracerebral hemorrhage, 2 with cerebral infarction, and 2 with TBI, and 3 were patients with UWS, including 2 with TBI and 1 with intracerebral hemorrhage. The only patient who showed CRS-R improvement during the sham period was a patient with TBI diagnosed as MCS. Overall, 13 of 17 patients with MCS showed a response during the rTMS period, whereas 6 of 23 patients with UWS showed a response during the rTMS period. By etiology, response during the rTMS period was observed in 10 of 17 patients with TBI, 7 of 20 patients with intracerebral hemorrhage, and 2 of 3 patients with cerebral infarction.

### 3.2 Network functional changes after intervention

Compared with the sham period, the rTMS period showed greater increases in ALFF at SMN.16, DMN.86, DMN.105, MRN.133, FPCN.189, SCN.223, and CN.245, while ALFF at VN.168 was significantly decreased (all *p* < 0.05; Figure 2D). Degree centrality (DC) increased at DMN.86 and SCN.232 during the rTMS period (*p* < 0.05; Figure 2E). For ReHo, SMN.14, SMN.16, DMN.124, and SCN.223 increased, whereas VN.170 decreased during the rTMS period (*p* < 0.05; Figure 2F). In addition, functional connectivity (FC) increased for DMN.86–CN.245, DMN.86–DMN.105, DMN.86–SCN.223, SCN.223–CN.246, DMN.79–MRN.133, DMN.82–FPCN.189, and DMN.107–SCN.222, while FC decreased for VN.155–VN.168 (all *p* < 0.05; Figure 2G). The MNI coordinates and AAL-based anatomical labels of all ROIs used in this study are provided in Supplementary material 1.

### 3.3 Explainable machine learning using network-change features to predict recovery

**Figure 3:**
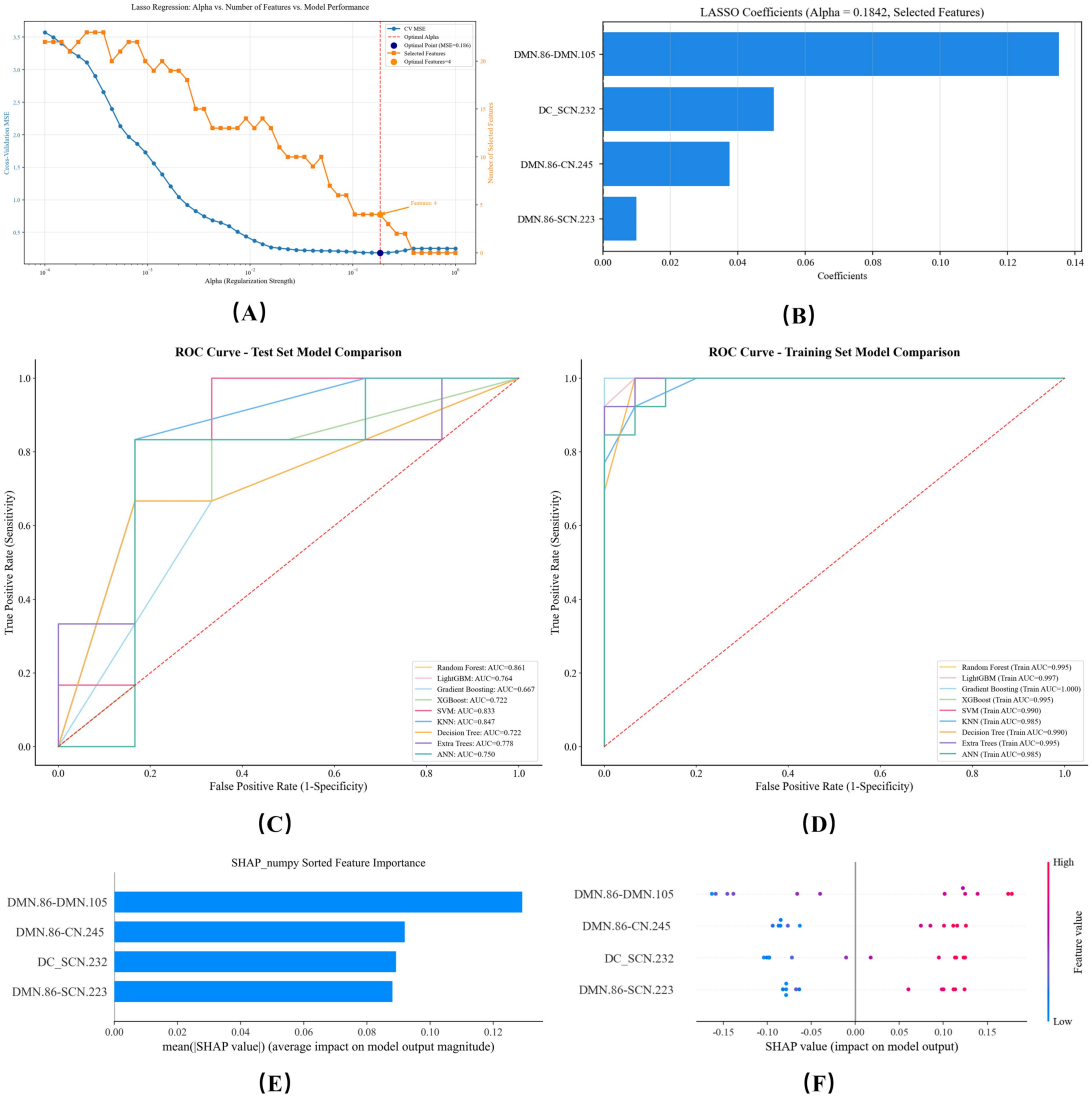
Explainable machine-learning identification of network changes associated with behavioral improvement during rTMS. Patients were categorized as responders or non-responders based on whether they showed behavioral improvement during the rTMS period (defined as a ≥ 1-point increase in any Coma Recovery Scale–Revised subscale relative to the period baseline). Period-related changes (*Δ*) in resting-state network metrics during rTMS were used as candidate features to characterize response. **(A)** LASSO feature selection from 23 rTMS-related change features; the optimal regularization parameter (*α* = 0.1842) yielded the best-performing sparse feature set. **(B)** Four selected features retained for model construction: functional connectivity (FC) of DMN.86–DMN.105, degree centrality (DC) of SCN.232, FC of DMN.86–CN.245, and FC of DMN.86–SCN.223. **(C,D)** Receiver operating characteristic (ROC) curves comparing nine classifiers; the random forest model achieved the highest discrimination in test-set (AUC = 0.861). **(E)** SHAP summary plot showing the relative contribution of each selected feature to response classification. **(F)** SHAP dependence plots illustrating that increases in DMN.86–SCN.223 FC and DMN.86–CN.245 FC were the dominant drivers of responder prediction. DMN, default mode network; SCN, subcortical network; CN, cerebellar network; FC, functional connectivity; DC, degree centrality; LASSO, least absolute shrinkage and selection operator; SHAP, Shapley additive explanations; AUC, area under the curve.

Patients were classified as responders or non-responders based on whether consciousness improved during the rTMS period, and change scores of network metrics across the period were used to build explainable machine learning models to identify recovery-linked network signatures. From 23 stimulation-related change features, LASSO regression further selected the optimal feature set, yielding the best performance at *α* = 0.1842 with four retained features (Figure 3A). These features were FC of DMN.86–DMN.105, DC of SCN.232, FC of DMN.86–CN.245, and FC of DMN.86–SCN.223 (Figure 3B). Among nine candidate models, the random forest model performed best (AUC = 0.861; Figures 3C,D). SHAP interpretation indicated that improvements in FC of DMN.86–SCN.223 and DMN.86–CN.245 contributed most prominently to predicting recovery (Figures 3E,F). Supplementary material 3 presents secondary performance metrics for model evaluation.

### 3.4 Baseline network differences between responders and non-responders

**Figure 4:**
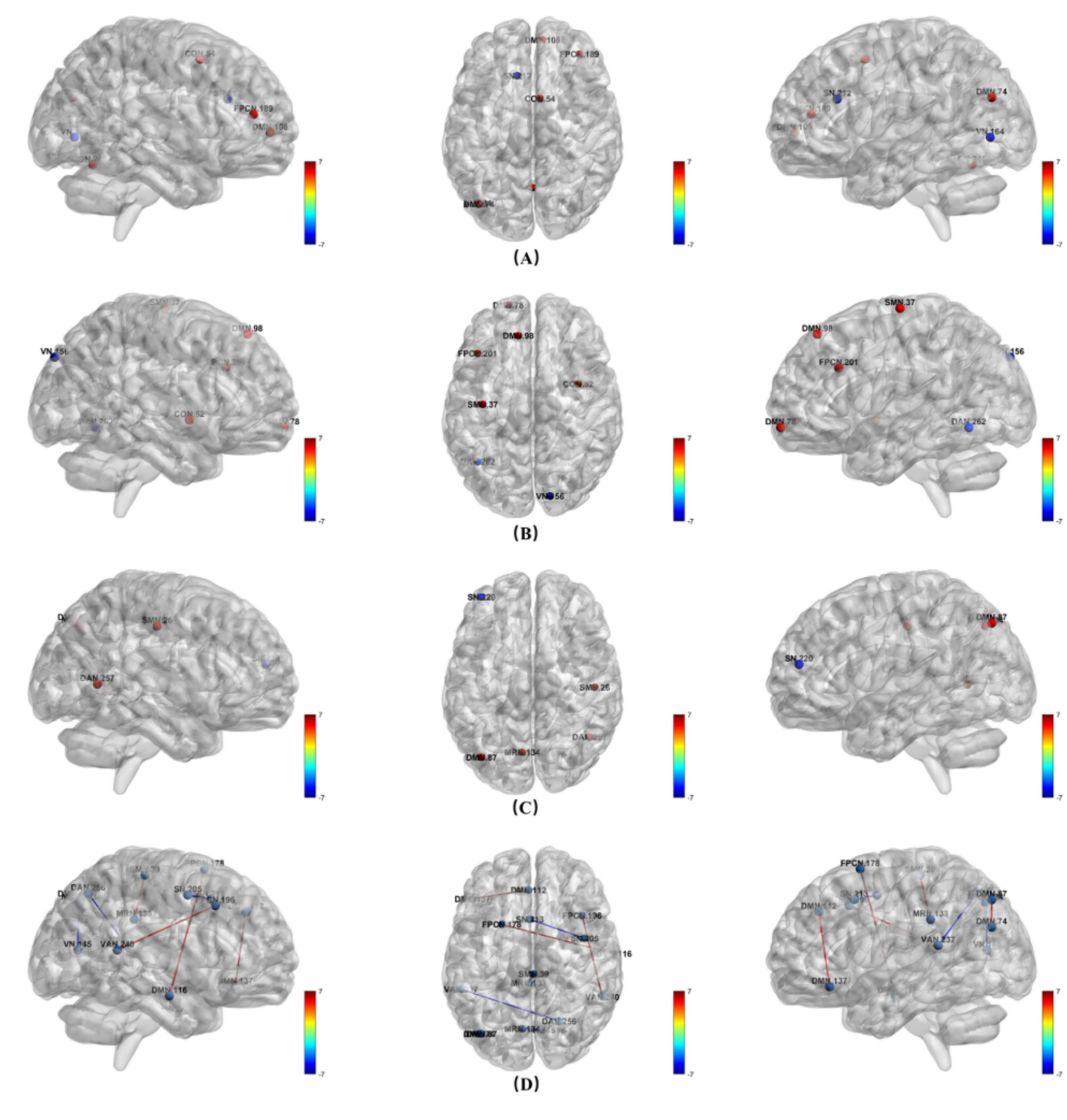
Baseline resting-state network features differentiate responders from non-responders to left angular gyrus rTMS. Patients were classified as responders or non-responders according to whether they demonstrated behavioral improvement during the rTMS period (defined as a ≥ 1-point increase in any Coma Recovery Scale–Revised subscale relative to the period baseline). Baseline resting-state fMRI metrics were compared between groups to identify pre-treatment network differences. **(A)** ALFF differences at baseline: responders showed higher ALFF in DMN.74, DMN.108, FPCN.189, CON.54, SN.212, and CN.246, and lower ALFF in VN.164. **(B)** Degree centrality (DC) differences at baseline: responders exhibited higher DC in SMN.26, DMN.87, MRN.134, and DAN.257, and lower DC in SN.220. **(C)** Regional homogeneity (ReHo) differences at baseline: responders showed higher ReHo in SMN.37, CON.52, DMN.78, DMN.98, and FPCN.201, and lower ReHo in VN.156 and DAN.262. **(D)** Functional connectivity (FC) differences at baseline: responders demonstrated higher FC for DMN.74–DMN.87, DMN.112–DMN.137, SMN.39–MRN.133, DMN.116–FPCN.178, and FPCN.196–VAN.240, and lower FC for MRN.134–VN.145, SN.205–SN.213, and VAN.237–DAN.256. For brain maps, warm colors indicate greater increases and cool colors indicate greater decreases during rTMS versus sham; lines denote FC differences (red, increased; blue, decreased). ALFF, amplitude of low-frequency fluctuations; ReHo, regional homogeneity; DC, degree centrality; FC, functional connectivity; DMN, default mode network; FPCN, frontoparietal control network; CON, cingulo-opercular network; SMN, sensorimotor network; MRN, memory retrieval network; CN, cerebellar network; DAN, dorsal attention network; VN, visual network; SN, salience network; VAN, ventral attention network.

Compared with non-responders (no improvement during the rTMS period), responders showed higher baseline ALFF at DMN.74, DMN.108, FPCN.189, CON.54, SN.212, and CN.246, and lower baseline ALFF at VN.164 (*p* < 0.05; Figure 4A). Responders also exhibited higher baseline DC at SMN.26, DMN.87, MRN.134, and DAN.257, but lower DC at SN.220 (*p* < 0.05; Figure 4B). Baseline ReHo was higher in responders at SMN.37, CON.52, DMN.78, DMN.98, and FPCN.201, and lower at VN.156 and DAN.262 (*p* < 0.05; Figure 4C). For FC, responders had higher baseline FC for DMN.74–DMN.87, DMN.112–DMN.137, SMN.39–MRN.133, DMN.116–FPCN.178, and FPCN.196–VAN.240, while lower baseline FC was observed for MRN.134–VN.145, SN.205–SN.213, and VAN.237–DAN.256 (*p* < 0.05; Figure 4D). The MNI coordinates and AAL-based anatomical labels of all ROIs used in this study are provided in Supplementary material 1.

### 3.5 Explainable machine learning using baseline features to predict recovery

**Figure 5:**
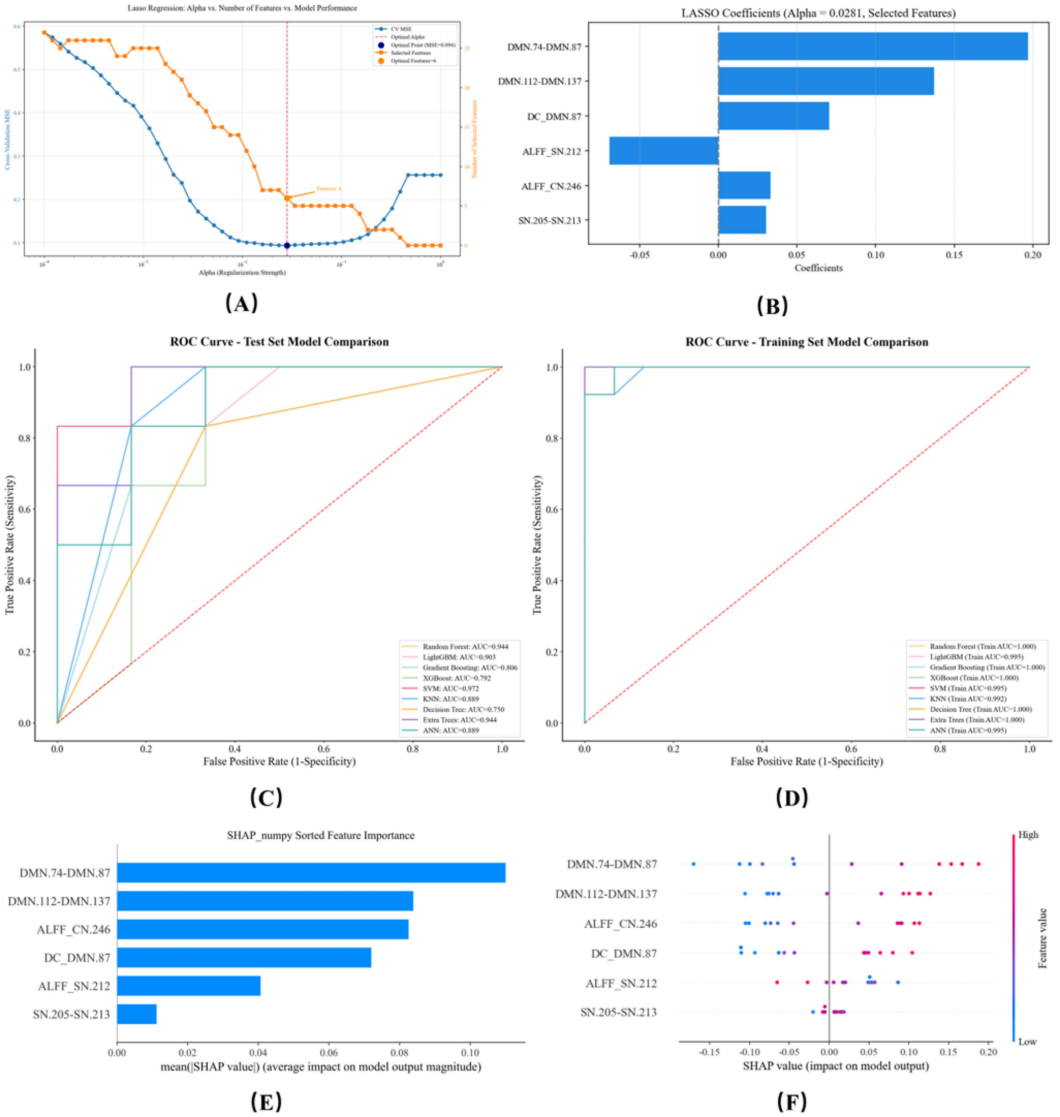
Explainable machine-learning prediction of behavioral improvement from baseline network features. Baseline resting-state fMRI features that differed between responders and non-responders (*n* = 27) were used to build explainable models to predict behavioral improvement during the rTMS period (response defined as a ≥ 1-point increase in any Coma Recovery Scale–Revised subscale relative to the period baseline). **(A)** LASSO feature selection from 27 baseline candidate features; the optimal regularization parameter (α = 0.0281) produced the best-performing sparse model. **(B)** Six retained baseline features: functional connectivity (FC) of DMN.74–DMN.87 and DMN.112–DMN.137, degree centrality (DC) of DMN.87, ALFF of SN.212 and CN.246, and FC of SN.205–SN.213. **(C,D)** Receiver operating characteristic (ROC) curves comparing nine classifiers; the support vector machine (SVM) model achieved the highest discriminative performance in test set (AUC = 0.972). **(E)** HAP summary plot ranking the contribution of the six baseline features to response prediction. **(F)** SHAP dependence plots indicating that higher DMN.74–DMN.87 FC, higher DMN.112–DMN.137 FC, and higher CN.246 ALFF were the strongest drivers of predicted response. DMN, default mode network; SN, salience network; CN, cerebellar network; FC, functional connectivity; DC, degree centrality; ALFF, amplitude of low-frequency fluctuations; LASSO, least absolute shrinkage and selection operator; SHAP, Shapley additive explanations; SVM, support vector machine; AUC, area under the curve.

From 27 baseline features showing group differences, LASSO regression identified the optimal model feature set at *α* = 0.0281, retaining six features (Figure 5A): FC of DMN.74–DMN.87, FC of DMN.112–DMN.137, DC of DMN.87, ALFF of SN.212, ALFF of CN.246, and FC of SN.205–SN.213 (Figure 5B). Among nine models, the support vector machine achieved the best performance (AUC = 0.972; Figures 5C,D). Feature attribution suggested that higher FC of DMN.74–DMN.87, higher FC of DMN.112–DMN.137, and higher ALFF of CN.246 were the most influential predictors of recovery (Figures 5E,F). Supplementary material 3 presents secondary performance metrics for model evaluation.

## 4 Discussion

This study showed that high-frequency rTMS targeting the left angular gyrus promoted observable behavioral improvement in a subset of patients with pDoC. This improvement was not limited to enhanced activity at the local stimulation target, but was accompanied by functional reorganization among the DMN, SCN, and cerebellar network-related nodes. More importantly, patients with relatively preserved intra-DMN connectivity at baseline were more likely to benefit from stimulation. This suggests that treatment response may depend on the modifiability and reintegration capacity of residual brain networks, rather than being determined solely by stimulation parameters (15).

From a clinical behavioral perspective, improvements after rTMS were mainly observed in the visual, motor, and auditory CRS-R subscales. This pattern is consistent with the stepwise nature of recovery commonly seen in patients with pDoC. In these patients, a slight improvement in a CRS-R subscale does not indicate complete recovery of consciousness. However, it usually suggests detectable changes in the processing of external stimuli, motor output pathways, or arousal regulation. The descriptive analysis by diagnosis and etiology provides additional clinical context. Although the sample size was not sufficient for formal subgroup testing, responses during the active rTMS period were observed in both MCS and UWS patients. Numerically, response was more frequent in patients with MCS than in those with UWS. This finding is clinically plausible, as patients with MCS usually retain more reproducible behavioral signs and a greater degree of residual network function than patients with UWS. However, this observation should be interpreted cautiously because the study was not powered to determine whether diagnostic category modified treatment response. Similarly, responses during the rTMS period were observed across different etiologies, including TBI, intracerebral hemorrhage, and cerebral infarction. This suggests that the behavioral effect of left angular gyrus rTMS may not be restricted to a single etiology. Nevertheless, the small number of patients in each etiological subgroup precludes any firm conclusion regarding etiology-specific responsiveness. Current guidelines also emphasize that clinical judgment in disorders of consciousness should not rely only on total scores. Instead, attention should be paid to changes in sensory input, motor output, and communication ability reflected by different subscales of standardized assessments (17). Therefore, defining treatment response based on CRS-R subscale improvement may provide a sensitive way to capture early or partial signs of recovery in this population. Nevertheless, such changes should be interpreted as improvement in consciousness-related behavior rather than full restoration of conscious awareness (18).

Previous studies of non-invasive brain stimulation have commonly selected the motor cortex, prefrontal cortex, or posterior parietal cortex as stimulation targets. The reported therapeutic effects have varied substantially, which may be related to the network location of the target, the residual connectivity of individual patients, and differences in stimulation protocols. Existing evidence suggests that stimulation of the angular gyrus can improve the level of consciousness in some patients with disorders of consciousness, providing direct support for the left angular gyrus as an intervention target (19). EEG studies of 20 Hz rTMS in patients with disorders of consciousness have also shown that high-frequency stimulation can alter brain functional activity, suggesting that it may act by increasing cortical excitability and network synchronization (20). More recently, a crossover, randomized, double-blind, sham-controlled study targeting the posterior parietal cortex further supported the potential value of posterior association cortices in the neuromodulation of pDoC (21). Compared with these studies, the present study used the left angular gyrus as a clearly defined stimulation target and combined resting-state fMRI with explainable machine learning. This allowed us to link clinical improvement to specific brain network changes, rather than focusing only on behavioral scale changes or a single imaging metric. Another study based on the perturbational complexity index also suggested that responsiveness to rTMS may be associated with brain network complexity and integrative capacity. This is consistent with our finding that baseline network status predicted treatment response (22).

In this study, DMN.86 corresponded to the left angular gyrus and represented the node containing the stimulation target. After rTMS, both ALFF and DC increased at this node. In addition, its FC with DMN.105, corresponding to the right medial superior frontal gyrus, SCN.223, corresponding to the left thalamus, and CN.245, corresponding to right cerebellar lobule VI, was enhanced. This pattern suggests that the effects of left angular gyrus stimulation were more consistent with network-level reintegration than with an isolated increase in local neural activity. The angular gyrus is located at the parieto-temporo-occipital junction and is an important region for multimodal information integration. It participates in the transformation among visual, auditory, somatosensory, semantic, and internal representations. Changes in its activity may therefore influence the entry of external stimuli into higher-order cognitive networks. From the perspective of the neural mechanisms of consciousness, conscious state depends not only on activation of individual brain regions, but also on dynamic, complex, and stable patterns of coordination across distributed brain areas (23). The enhanced connectivity between the left angular gyrus and the medial prefrontal DMN node observed in this study suggests that rTMS may facilitate network activity related to internal state monitoring, self-referential processing, and integration within higher-order association cortices (24). Previous studies of covert consciousness have also suggested that dynamic changes in the DMN may be associated with residual conscious processing capacity. This is in line with the high explanatory weight of DMN-related features for treatment response in the present study (25).

Changes in the SCN further support the interpretation that rTMS may modulate consciousness through cortico-thalamo-basal ganglia circuits. Supplementary material showed that SCN.223 was located in the left thalamus, SCN.222 in the right thalamus, and SCN.232 in the left putamen. In the present study, ALFF and ReHo increased in the left thalamus, DC increased in the left putamen, and FC between the left angular gyrus and left thalamus was enhanced. This connection was also one of the main features predicting behavioral improvement in the SHAP analysis. The thalamus is not merely a sensory relay structure. It is involved in cortical information integration, maintenance of arousal, and regulation of long-range cortical connectivity. Studies of recovery mechanisms in pDoC have repeatedly emphasized the interaction among the thalamus, striatum, and frontoparietal networks (15). In patients with pDoC, disrupted effective connectivity within and between the DMN and the anterior forebrain mesocircuit has been considered relevant to impaired maintenance of consciousness (26). Therefore, enhanced connectivity between the left angular gyrus and left thalamus may reflect improved information transfer between higher-order association cortex and arousal-regulating circuits after stimulation. The increased DC of the left putamen further suggests that basal ganglia-related circuits may contribute to treatment response, particularly in motor output, behavioral initiation, and gating of cortical activity. Findings from anesthesia studies showing reduced thalamocortical connectivity during loss of consciousness also support the importance of thalamocortical coupling in maintaining conscious state (26).

Cerebellar network-related changes also deserve attention. CN.245 corresponded to right cerebellar lobule VI, and CN.246 corresponded to cerebellar vermis VI. In this study, FC between the left angular gyrus and right cerebellar lobule VI increased, FC between the left thalamus and cerebellar vermis increased, and baseline ALFF in the cerebellar vermis was one of the important features predicting treatment response. Traditional research on disorders of consciousness has focused mainly on the frontoparietal cortex, thalamus, and brainstem ascending arousal system, while the cerebellum has often been viewed primarily as a motor regulatory structure. However, recent functional network studies indicate that lower-order sensorimotor networks and cerebellar connectivity may also influence the clinical presentation of patients with disorders of consciousness (27). The involvement of cerebellar lobule VI and vermis VI in the present study may be related to visual tracking, postural adjustment, motor preparation, and sensorimotor prediction. This may also help explain why CRS-R improvements were mainly observed in the visual, motor, and auditory subscales. Resting-state fMRI studies in chronic disorders of consciousness have suggested that the integrity of lower-order networks can support clinical assessment and stratification. This is consistent with the concurrent changes in sensorimotor, cerebellar, and visual network-related nodes observed in this study (28). However, whether enhanced cerebellar connectivity directly contributes to recovery of consciousness or reflects a downstream effect of improved cortico-thalamic network function remains to be further tested using longitudinal causal models or combined TMS-EEG approaches.

The decreases in visual network-related metrics should not be interpreted simply as visual functional impairment. After rTMS, ALFF decreased in VN.168, corresponding to the left middle occipital gyrus, ReHo decreased in VN.170, corresponding to the right calcarine cortex, and FC between VN.155, corresponding to the left superior occipital gyrus, and VN.168 also decreased. At the same time, behavioral improvement mainly involved the visual subscale. A plausible interpretation is that treatment response does not necessarily require a global increase in local synchronization or low-frequency amplitude within primary visual regions. Instead, it may reflect a shift of lower-order visual networks from relatively isolated local activity toward more effective coordination with higher-order association networks, the thalamus, and cerebellar networks. In other words, improvement in visual behavior may depend on whether visual information can be further integrated into frontoparietal and default mode networks, rather than on enhanced occipital activity alone. Similarly, studies of language and higher-order auditory processing have suggested that patients with disorders of consciousness may retain sensory and semantic processing at different depths. Whether these capacities can be expressed as overt behavior depends on the integrative state of broader networks (29). Therefore, the reduction in local visual network metrics in this study is not necessarily inconsistent with improvement in the clinical visual subscale. Rather, it indicates that the direction of change in a single brain region should be interpreted within the context of whole-brain network organization.

Baseline feature analysis further indicated that treatment response was closely related to pre-intervention network reserve. The key baseline features selected by the explainable model included FC of DMN.74-DMN.87, FC of DMN.112-DMN.137, DC of DMN.87, ALFF of SN.212, ALFF of CN.246, and FC of SN.205-SN.213. Among these nodes, DMN.74 was located in the left middle occipital gyrus, DMN.87 in the left inferior parietal lobule, DMN.112 in the left medial superior frontal gyrus, and DMN.137 in the left middle frontal gyrus. Together, these nodes point to residual connectivity among posterior parietal regions, occipitoparietal association areas, and medial prefrontal regions within the DMN. In patients with pDoC, the DMN is not only a core network in consciousness research, but also an important candidate marker for distinguishing levels of consciousness and identifying covert consciousness (25). Therefore, patients with stronger intra-DMN connectivity at baseline may retain a certain degree of network plasticity and information integration capacity, making them more likely to show detectable behavioral improvement after high-frequency stimulation of the left angular gyrus.

In addition to DMN features, baseline ALFF of SN.212, corresponding to the left anterior cingulate cortex, and FC of SN.205-SN.213 were also included in the model. This suggests that the salience network and motor-related cingulate regions may influence treatment response. The anterior cingulate cortex participates in conflict monitoring, motivational drive, behavioral initiation, and switching between internal and external stimuli. These functions may be relevant for translating internal neural activity into observable responses in patients with disorders of consciousness. Notably, SN.205 was located in the right precentral gyrus and SN.213 in the left middle cingulate cortex. The direction of this connection in the model was not fully consistent with other favorable features, suggesting that stronger local motor-cingulate coupling may not necessarily indicate greater recovery potential. It may also reflect non-specific motor circuit activity or abnormal network coupling. Reviews of functional networks have suggested that the core disturbance in pDoC is not simply weakening of a single network, but an imbalance in integration and segregation across multiple large-scale networks (27). Thus, the baseline model in this study should be interpreted more as a tool for stratifying network state than as a simple linear judgment that higher values of a given metric are always better.

The main role of explainable machine learning in this study was to identify network features associated with recovery. The random forest model based on stimulation-period change features showed that left angular gyrus-left thalamus connectivity, left angular gyrus-right cerebellar lobule VI connectivity, left angular gyrus-right medial superior frontal gyrus connectivity, and DC of the left putamen together formed a network profile associated with behavioral improvement. In contrast, the SVM model based on baseline features indicated that pre-treatment intra-DMN connectivity and spontaneous activity of the cerebellar vermis contributed substantially to response prediction. These two models do not suggest conflicting mechanisms. The former reflects what type of network reconfiguration occurred after stimulation, whereas the latter reflects which patients were more likely to undergo such reconfiguration. This distinction has clinical relevance, because patients with pDoC are highly heterogeneous. Even with the same stimulation target and parameters, treatment effects may differ according to the residual network architecture. It should also be emphasized that a high AUC indicates good discrimination within the present sample, but should not be interpreted as evidence that the model is already mature enough for clinical prediction. External cohort validation remains necessary.

The scientific value of this study lies in linking the clinical effects of left angular gyrus rTMS with interpretable brain network mechanisms, thereby providing testable hypotheses for individualized neuromodulation in pDoC. The findings suggest that the left angular gyrus, as a multimodal association node within the DMN, may be a suitable stimulation target for patients with preserved residual DMN connectivity. Baseline network features, particularly intra-DMN connectivity and spontaneous activity in the cerebellar vermis, may help identify patients who are more likely to benefit from treatment. This approach may support a shift from a uniform stimulation protocol toward network state-based individualized intervention.

Several limitations should be acknowledged. First, this was a single-center study, and the sample size still limited the ability to conduct subgroup analyses. In particular, differences among patients with different etiologies, diagnostic levels, and disease durations could not be fully examined. Second, treatment response was defined as a 1-point increase in any CRS-R subscale. This definition is useful for capturing subtle but clinically meaningful changes in patients with pDoC, but it may also be influenced by diurnal fluctuation, fatigue, and the assessment window. Third, the stimulation target was localized to P5 using the 10–20 system. This approach is simple and clinically feasible, but it cannot fully correct for individual differences in brain structure. Future studies may use individualized MRI-guided neuronavigation to improve target consistency. Fourth, the machine learning models lacked independent external validation. In particular, when a baseline prediction model achieves a high AUC in a small sample, overfitting and the influence of sample distribution should be carefully considered. Future studies should validate these models in larger multicenter cohorts and integrate prediction results into real clinical decision-making pathways to assess their reproducibility and clinical utility.

## Data Availability

The raw data supporting the conclusions of this article will be made available by the authors, without undue reservation.
