# Supplementary material for: Effect and mechanism of repetitive transcranial magnetic stimulation of angular gyrus on patients with consciousness disorder
Source: Front Neurol. 2026 Jun 17;17:1868819. doi: 10.3389/fneur.2026.1868819 (PMC13318575; doi:10.3389/fneur.2026.1868819)
Supplement: Supplementary file 1 [file Table_1.DOCX]

Supplementary Material

**Supplementary Material** **1. Power-264 atlas description and ROI coordinates with AAL anatomical labels**

The present study used the Power-264 functional atlas, which defines a brain-wide graph of 264 putative functional areas (nodes) derived by merging meta-analytic ROIs and rs-fcMRI fc-mapping ROIs. The original atlas construction merged 151 non-overlapping meta-analytic ROIs with 193 non-overlapping fc-mapping ROIs to yield 264 independent ROIs, modeled as spherical ROIs. The atlas was published in the leading neuroscience journal Neuron and has since been widely adopted and validated, supporting its reproducibility across studies (Power JD, Cohen AL, Nelson SM, et al. Functional network organization of the human brain. Neuron. 2011;72:665-678. doi:10.1016/j.neuron.2011.09.006).

In our analyses, each ROI was labeled using the convention . (e.g., DMN.86), where the network assignment follows the Power-264 functional system labels and nodeID corresponds to the Power-264 node index used in our processing pipeline. AAL anatomical labels were assigned using the Automated Anatomical Labeling (AAL) framework. Table S1 lists the Power-264 nodes that were reported in the main analyses (i.e., nodes showing significant effects and entering prediction models) together with their MNI coordinates and AAL-based anatomical labels.

Table S1. Coordinates and anatomical locations of brain network ROIs reported in this study

| ROI | MNI x | MNI y | MNI z | AAL anatomical location |
| --- | --- | --- | --- | --- |
| SMN.14 | -14 | -18 | 40 | Cingulum Mid L |
| SMN.16 | 10 | -2 | 45 | Cingulum Mid R |
| SMN.26 | 50 | -20 | 42 | Postcentral R |
| SMN.37 | -38 | -15 | 69 | Precentral L |
| SMN.39 | 2 | -28 | 60 | Paracentral Lobule R |
| CON.52 | 37 | 1 | -4 | Insula R |
| CON.54 | 7 | 8 | 51 | Supp Motor Area R |
| DMN.74 | -41 | -75 | 26 | Occipital Mid L |
| DMN.78 | -18 | 63 | -9 | Frontal Sup Orb L |
| DMN.79 | -46 | -61 | 21 | Temporal Mid L |
| DMN.82 | 46 | 16 | -30 | Temporal Pole Mid R |
| DMN.86 | -44 | -65 | 35 | Angular L |
| DMN.87 | -39 | -75 | 44 | Parietal inf L |
| DMN.98 | -10 | 39 | 52 | Frontal Sup Medial L |
| DMN.105 | 6 | 54 | 16 | Frontal Sup Medial R |
| DMN.107 | -7 | 51 | -1 | Cingulum Ant L |
| DMN.108 | 9 | 54 | 3 | Frontal Sup Medial R |
| DMN.112 | -2 | 38 | 36 | Frontal Sup Medial L |
| DMN.116 | 65 | -12 | -19 | Temporal Mid R |
| DMN.124 | -26 | -40 | -8 | ParaHippocampal L |
| MRN.133 | -2 | -35 | 31 | Cingulum Post L |
| MRN.134 | -7 | -71 | 42 | Precuneus L |
| DMN.137 | -46 | 31 | -13 | Frontal Mid L |
| VN.145 | 8 | -72 | 11 | Calcarine R |
| VN.155 | -14 | -91 | 31 | Occipital Sup L |
| VN.156 | 15 | -87 | 37 | Occipital Sup R |
| VN.164 | -42 | -74 | 0 | Occipital Mid L |
| VN.168 | -40 | -88 | -6 | Occipital Mid L |
| VN.170 | 6 | -81 | 6 | Calcarine R |
| FPCN.178 | -23 | 11 | 64 | Putamen L |
| FPCN.189 | 38 | 43 | 15 | Frontal Mid R |
| FPCN.196 | 40 | 18 | 40 | Frontal Mid R |
| FPCN.201 | -42 | 25 | 30 | Frontal Inf Tri L |
| SN.205 | 42 | 0 | 47 | Precentral R |
| SN.212 | -11 | 26 | 25 | Cingulum Ant L |
| SN.213 | -1 | 15 | 44 | Cingulum Mid L |
| SN.220 | -39 | 51 | 17 | Frontal Mid L |
| SCN.222 | 6 | -24 | 0 | Thalamus R |
| SCN.223 | -2 | -13 | 12 | Thalamus L |
| SCN.232 | -31 | -11 | 0 | Putamen L |
| VAN.237 | -55 | -40 | 14 | Temporal Sup L |
| VAN.240 | 56 | -46 | 11 | Temporal Mid R |
| CN.245 | 22 | -58 | -23 | Cerebelum6 R |
| CN.246 | 1 | -62 | -18 | Vermis 6 |
| DAN.257 | 46 | -59 | 4 | Temporal Mid R |
| DAN.256 | 22 | -65 | 48 | Occipital Sup R |
| DAN.262 | -42 | -60 | -9 | Temporal Inf L |

Notes. Coordinates are in MNI space (mm). AAL anatomical labels were assigned using the Automated Anatomical Labeling (AAL) framework. Network abbreviations: DMN, default mode network; FPCN, frontoparietal control network; CON, cingulo-opercular network; SMN, sensorimotor network; MRN, memory retrieval network; CN, cerebellar network; DAN, dorsal attention network; VN, visual network; SN, salience network; SCN, subcortical network; VAN, ventral attention network. ROI labels follow the convention . used throughout the manuscript; node definitions are based on the Power-264 atlas.

**Supplementary Material 2. Baseline and end-of-cycle CRS-R subscale scores across two treatment cycles in all participants from both groups**

S2.1 CRS-R subscale scores in the rTMS-sham group

| Cycle 1 baseline CRS-R subscale scores | | | | | |
| --- | --- | --- | --- | --- | --- |
| Auditory | Visual | Motor | Oromotor/Verbal | Communication | Arousal |
| 2 | 2 | 1 | 1 | 0 | 2 |
| 0 | 0 | 1 | 0 | 0 | 1 |
| 2 | 3 | 4 | 1 | 0 | 2 |
| 2 | 2 | 2 | 2 | 0 | 2 |
| 2 | 3 | 5 | 2 | 0 | 2 |
| 1 | 1 | 2 | 2 | 0 | 2 |
| 2 | 3 | 2 | 2 | 0 | 2 |
| 2 | 3 | 4 | 2 | 0 | 2 |
| 0 | 1 | 1 | 0 | 0 | 1 |
| 0 | 0 | 2 | 0 | 0 | 1 |
| 1 | 1 | 1 | 1 | 0 | 2 |
| 3 | 3 | 5 | 2 | 0 | 2 |
| 1 | 1 | 1 | 1 | 0 | 2 |
| 0 | 1 | 1 | 0 | 0 | 1 |
| 1 | 0 | 2 | 0 | 0 | 1 |
| 2 | 2 | 4 | 2 | 0 | 2 |
| 1 | 1 | 2 | 1 | 0 | 1 |
| 3 | 3 | 5 | 2 | 0 | 2 |
| 0 | 0 | 1 | 0 | 0 | 1 |
| 0 | 0 | 1 | 0 | 0 | 1 |
| Cycle 1 end CRS-R subscale scores | | | | | |
| Auditory | Visual | Motor | Oromotor/Verbal | Communication | Arousal |
| 2 | 2 | 3 | 1 | 0 | 2 |
| 0 | 0 | 1 | 0 | 0 | 1 |
| 2 | 3 | 5 | 1 | 0 | 2 |
| 2 | 2 | 3 | 2 | 0 | 2 |
| 3 | 4 | 5 | 2 | 0 | 2 |
| 1 | 1 | 2 | 2 | 0 | 2 |
| 2 | 3 | 2 | 2 | 0 | 3 |
| 2 | 4 | 4 | 2 | 0 | 2 |
| 0 | 1 | 1 | 0 | 0 | 1 |
| 0 | 0 | 2 | 0 | 0 | 1 |
| 1 | 1 | 1 | 1 | 0 | 2 |
| 4 | 4 | 5 | 2 | 0 | 2 |
| 1 | 1 | 1 | 1 | 0 | 2 |
| 0 | 1 | 1 | 0 | 0 | 1 |
| 1 | 1 | 2 | 0 | 0 | 1 |
| 3 | 2 | 4 | 2 | 0 | 2 |
| 1 | 1 | 2 | 1 | 0 | 1 |
| 3 | 3 | 5 | 2 | 0 | 2 |
| 0 | 1 | 1 | 0 | 0 | 1 |
| 0 | 0 | 1 | 0 | 0 | 1 |
| Cycle 2 baseline CRS-R subscale scores | | | | | |
| Auditory | Visual | Motor | Oromotor/Verbal | Communication | Arousal |
| 2 | 2 | 3 | 1 | 0 | 2 |
| 0 | 0 | 1 | 0 | 0 | 1 |
| 2 | 3 | 5 | 1 | 0 | 2 |
| 2 | 2 | 3 | 2 | 0 | 2 |
| 3 | 4 | 5 | 2 | 0 | 2 |
| 1 | 1 | 2 | 2 | 0 | 2 |
| 2 | 3 | 2 | 2 | 0 | 3 |
| 2 | 4 | 4 | 2 | 0 | 2 |
| 0 | 1 | 1 | 0 | 0 | 1 |
| 0 | 0 | 2 | 0 | 0 | 1 |
| 1 | 1 | 1 | 1 | 0 | 2 |
| 4 | 4 | 5 | 2 | 0 | 2 |
| 1 | 1 | 1 | 1 | 0 | 2 |
| 0 | 1 | 1 | 0 | 0 | 1 |
| 1 | 1 | 2 | 0 | 0 | 1 |
| 3 | 2 | 4 | 2 | 0 | 2 |
| 1 | 1 | 2 | 1 | 0 | 1 |
| 3 | 3 | 5 | 2 | 0 | 2 |
| 0 | 1 | 1 | 0 | 0 | 1 |
| 0 | 0 | 1 | 0 | 0 | 1 |
| Cycle 2 end CRS-R subscale scores | | | | | |
| Auditory | Visual | Motor | Oromotor/Verbal | Communication | Arousal |
| 2 | 2 | 3 | 1 | 0 | 2 |
| 0 | 0 | 1 | 0 | 0 | 1 |
| 2 | 3 | 5 | 1 | 0 | 2 |
| 2 | 3 | 3 | 2 | 0 | 2 |
| 3 | 4 | 5 | 2 | 0 | 2 |
| 1 | 1 | 2 | 2 | 0 | 2 |
| 2 | 3 | 3 | 2 | 0 | 3 |
| 2 | 4 | 4 | 2 | 0 | 2 |
| 0 | 1 | 1 | 0 | 0 | 1 |
| 0 | 0 | 2 | 0 | 0 | 1 |
| 1 | 1 | 1 | 1 | 0 | 2 |
| 4 | 4 | 5 | 2 | 0 | 2 |
| 1 | 1 | 1 | 1 | 0 | 2 |
| 0 | 1 | 1 | 0 | 0 | 1 |
| 1 | 1 | 2 | 0 | 0 | 1 |
| 3 | 3 | 4 | 2 | 0 | 2 |
| 1 | 1 | 2 | 1 | 0 | 1 |
| 3 | 3 | 5 | 2 | 0 | 2 |
| 0 | 1 | 1 | 0 | 0 | 1 |
| 0 | 0 | 1 | 0 | 0 | 1 |

S2.2 CRS-R subscale scores in the sham- rTMS group

| Cycle 1 baseline CRS-R subscale scores | | | | | |
| --- | --- | --- | --- | --- | --- |
| Auditory | Visual | Motor | Oromotor/Verbal | Communication | Arousal |
| 2 | 3 | 4 | 1 | 0 | 2 |
| 0 | 1 | 1 | 0 | 0 | 1 |
| 1 | 3 | 3 | 2 | 0 | 2 |
| 0 | 0 | 4 | 0 | 0 | 1 |
| 0 | 1 | 2 | 0 | 0 | 2 |
| 2 | 3 | 5 | 1 | 0 | 2 |
| 1 | 1 | 2 | 0 | 0 | 1 |
| 1 | 1 | 2 | 0 | 0 | 1 |
| 0 | 0 | 1 | 1 | 0 | 2 |
| 1 | 1 | 1 | 1 | 0 | 2 |
| 1 | 1 | 2 | 1 | 0 | 2 |
| 1 | 3 | 5 | 2 | 0 | 2 |
| 1 | 0 | 2 | 0 | 0 | 2 |
| 2 | 3 | 4 | 2 | 0 | 2 |
| 3 | 3 | 4 | 2 | 0 | 2 |
| 0 | 0 | 1 | 0 | 0 | 1 |
| 3 | 3 | 4 | 1 | 0 | 2 |
| 1 | 0 | 2 | 0 | 0 | 1 |
| 3 | 3 | 2 | 2 | 0 | 2 |
| 0 | 0 | 1 | 0 | 0 | 1 |
| Cycle 1 end CRS-R subscale scores | | | | | |
| Auditory | Visual | Motor | Oromotor/Verbal | Communication | Arousal |
| 2 | 3 | 4 | 1 | 0 | 2 |
| 0 | 1 | 1 | 0 | 0 | 1 |
| 1 | 3 | 3 | 2 | 0 | 2 |
| 0 | 0 | 4 | 0 | 0 | 1 |
| 0 | 1 | 2 | 0 | 0 | 2 |
| 2 | 3 | 5 | 1 | 0 | 2 |
| 1 | 1 | 2 | 0 | 0 | 1 |
| 1 | 1 | 2 | 0 | 0 | 1 |
| 0 | 0 | 1 | 1 | 0 | 2 |
| 1 | 1 | 1 | 1 | 0 | 2 |
| 1 | 1 | 2 | 1 | 0 | 2 |
| 1 | 3 | 5 | 2 | 0 | 2 |
| 1 | 0 | 2 | 0 | 0 | 2 |
| 2 | 4 | 4 | 2 | 0 | 2 |
| 3 | 3 | 4 | 2 | 0 | 2 |
| 0 | 0 | 1 | 0 | 0 | 1 |
| 3 | 3 | 4 | 1 | 0 | 2 |
| 1 | 0 | 2 | 0 | 0 | 1 |
| 3 | 3 | 2 | 2 | 0 | 2 |
| 0 | 0 | 1 | 0 | 0 | 1 |
| Cycle 2 baseline CRS-R subscale scores | | | | | |
| Auditory | Visual | Motor | Oromotor/Verbal | Communication | Arousal |
| 2 | 3 | 4 | 1 | 0 | 2 |
| 0 | 1 | 1 | 0 | 0 | 1 |
| 1 | 3 | 3 | 2 | 0 | 2 |
| 0 | 0 | 4 | 0 | 0 | 1 |
| 0 | 1 | 2 | 0 | 0 | 2 |
| 2 | 3 | 5 | 1 | 0 | 2 |
| 1 | 1 | 2 | 0 | 0 | 1 |
| 1 | 1 | 2 | 0 | 0 | 1 |
| 0 | 0 | 1 | 1 | 0 | 2 |
| 1 | 1 | 1 | 1 | 0 | 2 |
| 1 | 1 | 2 | 1 | 0 | 2 |
| 1 | 3 | 5 | 2 | 0 | 2 |
| 1 | 0 | 2 | 0 | 0 | 2 |
| 2 | 4 | 4 | 2 | 0 | 2 |
| 3 | 3 | 4 | 2 | 0 | 2 |
| 0 | 0 | 1 | 0 | 0 | 1 |
| 3 | 3 | 4 | 1 | 0 | 2 |
| 1 | 0 | 2 | 0 | 0 | 1 |
| 3 | 3 | 2 | 2 | 0 | 2 |
| 0 | 0 | 1 | 0 | 0 | 1 |
| Cycle 2 end CRS-R subscale scores | | | | | |
| Auditory | Visual | Motor | Oromotor/Verbal | Communication | Arousal |
| 4 | 3 | 4 | 1 | 0 | 2 |
| 0 | 1 | 1 | 0 | 0 | 1 |
| 2 | 3 | 3 | 2 | 0 | 2 |
| 0 | 0 | 4 | 0 | 0 | 1 |
| 0 | 1 | 3 | 0 | 0 | 2 |
| 2 | 4 | 5 | 1 | 0 | 2 |
| 1 | 1 | 2 | 0 | 0 | 1 |
| 1 | 1 | 2 | 0 | 0 | 1 |
| 1 | 0 | 1 | 1 | 0 | 2 |
| 1 | 1 | 1 | 1 | 0 | 2 |
| 1 | 1 | 2 | 1 | 0 | 2 |
| 1 | 4 | 5 | 2 | 0 | 2 |
| 1 | 1 | 2 | 0 | 0 | 2 |
| 2 | 4 | 4 | 2 | 1 | 2 |
| 3 | 3 | 4 | 2 | 0 | 3 |
| 0 | 0 | 1 | 0 | 0 | 1 |
| 3 | 3 | 4 | 2 | 0 | 2 |
| 1 | 0 | 2 | 0 | 0 | 1 |
| 3 | 3 | 2 | 2 | 0 | 2 |
| 0 | 0 | 1 | 0 | 0 | 1 |

**Supplementary Material 3. Model evaluation and explainability for prediction analyses**

Two explainable machine-learning analyses were conducted to (i) relate stimulation-period network changes to behavioral improvement and (ii) identify baseline network features associated with response. Model performance was primarily assessed using ROC–AUC, complemented by threshold-dependent metrics derived from the confusion matrix. Feature contributions were interpreted using SHAP values.

S3.1 Model evaluation

S3.1.1 ROC curve and AUC

Receiver operating characteristic (ROC) curves summarize discrimination performance across decision thresholds by plotting the true positive rate (TPR) against the false positive rate (FPR). Curves closer to the upper-left corner indicate better discrimination. The area under the ROC curve (AUC) ranges from 0 to 1; values closer to 1 indicate stronger predictive performance, whereas AUC = 0.5 indicates chance-level discrimination. Accordingly, AUC was used as the primary metric for model comparison in this study.

S3.1.2 Confusion matrix and threshold-dependent metrics

For binary classification, predictions were summarized using a confusion matrix. The positive class was defined as behavioral improvement during the rTMS period (Responder = 1) and the negative class as no improvement (Non-responder = 0). Let TP, TN, FP, and FN denote true positives, true negatives, false positives, and false negatives, respectively. Metrics were computed as follows: Accuracy = (TP + TN) / (TP + TN + FP + FN); Precision (PPV) = TP / (TP + FP); Recall (Sensitivity) = TP / (TP + FN); Specificity = TN / (TN + FP); Negative predictive value (NPV) = TN / (TN + FN); F1-score = 2 × (Precision × Recall) / (Precision + Recall); Cohen’s kappa was additionally used to quantify agreement beyond chance.

S3.2 Model explainability (SHAP)

To interpret the contribution of each feature to model predictions, SHAP (SHapley Additive exPlanations) values were used to quantify feature importance at both the population and individual levels. SHAP values represent the marginal contribution of each feature to the model output relative to the model baseline; positive SHAP values indicate that the feature increases the predicted probability of response, whereas negative values indicate a decrease. Feature rankings were obtained by sorting SHAP values.

S3.3 The results of Secondary performance metrics for model evaluation

To complement the primary discrimination results reported in the main text and figures (ROC–AUC), we provide secondary, threshold-dependent performance metrics derived from the confusion matrix for each candidate classifier. These results are reported separately for the training set (Train) and an independent held-out test set (Test) for the two prediction settings: (i) models built from intervention-related network change features (Δ) and (ii) models built from baseline network features. The purpose of these supplementary results is to offer additional context on classification behavior at the chosen operating threshold, rather than to redefine model ranking, which was based on ROC–AUC.

Table S3A. Models using intervention-related network change features (Δ)

| **Test set** | ANN | DT | ET | GBM | KNN | LightGBM | RF | SVM | XGBoost |
| --- | --- | --- | --- | --- | --- | --- | --- | --- | --- |
| Accuracy | 0.833 | 0.667 | 0.833 | 0.667 | 0.833 | 0.667 | 0.833 | 0.833 | 0.750 |
| Sensitivity | 0.833 | 0.667 | 0.833 | 0.667 | 0.833 | 0.667 | 0.833 | 0.833 | 0.667 |
| Specificity | 0.833 | 0.667 | 0.833 | 0.667 | 0.833 | 0.667 | 0.833 | 0.833 | 0.833 |
| PPV | 0.833 | 0.667 | 0.833 | 0.667 | 0.833 | 0.667 | 0.833 | 0.833 | 0.800 |
| NPV | 0.833 | 0.667 | 0.833 | 0.667 | 0.833 | 0.667 | 0.833 | 0.833 | 0.667 |
| F1 score | 0.833 | 0.667 | 0.833 | 0.667 | 0.833 | 0.667 | 0.833 | 0.833 | 0.727 |
| Cohen’s κ | 0.667 | 0.333 | 0.667 | 0.333 | 0.667 | 0.333 | 0.667 | 0.667 | 0.500 |

| **Train set** | ANN | DT | ET | GBM | KNN | LightGBM | RF | SVM | XGBoost |
| --- | --- | --- | --- | --- | --- | --- | --- | --- | --- |
| Accuracy | 0.929 | 0.964 | 0.929 | 1.000 | 0.929 | 0.964 | 0.929 | 0.929 | 0.929 |
| Sensitivity | 0.923 | 1.000 | 0.923 | 1.000 | 0.923 | 1.000 | 0.923 | 0.923 | 0.923 |
| Specificity | 0.933 | 0.933 | 0.933 | 1.000 | 0.933 | 0.933 | 0.933 | 0.933 | 0.933 |
| PPV | 0.923 | 0.929 | 0.923 | 1.000 | 0.923 | 0.929 | 0.923 | 0.923 | 0.923 |
| NPV | 0.923 | 1.000 | 0.923 | 1.000 | 0.923 | 1.000 | 0.923 | 0.923 | 0.923 |
| F1 score | 0.923 | 0.963 | 0.923 | 1.000 | 0.923 | 0.963 | 0.923 | 0.923 | 0.923 |
| Cohen’s κ | 0.856 | 0.929 | 0.856 | 1.000 | 0.856 | 0.929 | 0.856 | 0.856 | 0.856 |

Table S3B. Models using baseline network features

| **Test set** | ANN | DT | ET | GBM | KNN | LightGBM | RF | SVM | XGBoost |
| --- | --- | --- | --- | --- | --- | --- | --- | --- | --- |
| Accuracy | 0.833 | 0.833 | 0.833 | 0.833 | 0.833 | 0.667 | 0.833 | 0.833 | 0.667 |
| Sensitivity | 0.833 | 0.667 | 0.833 | 0.667 | 0.833 | 0.833 | 0.833 | 0.833 | 0.833 |
| Specificity | 0.833 | 0.714 | 0.833 | 0.714 | 0.833 | 0.800 | 0.833 | 0.833 | 0.800 |
| PPV | 0.833 | 0.833 | 0.833 | 0.833 | 0.833 | 0.667 | 0.833 | 0.833 | 0.667 |
| NPV | 0.833 | 0.769 | 0.833 | 0.769 | 0.833 | 0.727 | 0.833 | 0.833 | 0.727 |
| F1 score | 0.667 | 0.500 | 0.667 | 0.500 | 0.667 | 0.500 | 0.667 | 0.667 | 0.500 |
| Cohen’s κ | 0.833 | 0.750 | 0.833 | 0.750 | 0.833 | 0.750 | 0.833 | 0.833 | 0.750 |

| **Train set** | ANN | DT | ET | GBM | KNN | LightGBM | RF | SVM | XGBoost |
| --- | --- | --- | --- | --- | --- | --- | --- | --- | --- |
| Accuracy | 0.929 | 1.000 | 1.000 | 1.000 | 0.929 | 0.964 | 1.000 | 0.964 | 0.964 |
| Sensitivity | 0.923 | 1.000 | 1.000 | 1.000 | 0.923 | 0.923 | 1.000 | 0.923 | 0.923 |
| Specificity | 0.933 | 1.000 | 1.000 | 1.000 | 0.933 | 1.000 | 1.000 | 1.000 | 1.000 |
| PPV | 0.923 | 1.000 | 1.000 | 1.000 | 0.923 | 1.000 | 1.000 | 1.000 | 1.000 |
| NPV | 0.923 | 1.000 | 1.000 | 1.000 | 0.923 | 0.923 | 1.000 | 0.923 | 0.923 |
| F1 score | 0.923 | 1.000 | 1.000 | 1.000 | 0.923 | 0.960 | 1.000 | 0.960 | 0.960 |
| Cohen’s κ | 0.856 | 1.000 | 1.000 | 1.000 | 0.856 | 0.928 | 1.000 | 0.928 | 0.928 |

Notes: These are secondary, threshold-dependent metrics. The primary model ranking was based on ROC–AUC, reported in the main text/figures for the best-performing models (random forest for Δ-feature prediction; support vector machine for baseline-feature prediction). Train denotes performance on the training split after model fitting; Test denotes performance on an independent held-out test split.
